# Mucormycosis of Neck a Rare Presentation

**DOI:** 10.1007/s12070-020-02006-5

**Published:** 2020-08-05

**Authors:** Feroze Khan Kancharu, Vineeth Viswam, P. Sreeram, Anup Warrier, Aparna Karunakaran

**Affiliations:** 1 Department of ENT, Aster Medcity, Kochi, Kerala India; 2 Department of Infectious Disease, Aster Medcity, Kochi, Kerala India

**Keywords:** Mucormycosis, Mastoiditis, Lemierre syndrome

## Abstract

Mucormycosis is a dreaded clinical entity caused by filamentous fungi of the order Mucorales mainly affecting immunocompromised individuals. Usually seen involving paranasal sinuses, orbit, lungs and gastrointestinal system, it is extremely rare in other areas. Herein we report a rare presentation of Mucormycosis affecting the ear and neck where early detection, timely intervention, multidisciplinary involvement and judicious use of local antibiotics apart from the mainstay treatment regimen like surgical debridement and intravenous Amphotericin B has saved the patient and given him a reasonably good quality of life devoid of morbidity.

## Introduction

Mucormycosis was first described by Paultauf in 1885 [1]. It is a rare fungal infection affecting immunocompromised patients mostly diabetics. The common varieties of mucormycosis encountered in clinical practice are those involving the paranasal sinuses (39%), lungs (24%), skin (19%), brain (9%), and gastrointestinal system(7%) [2]. Apart from this, there are other types like cutaneous which are extremely rare.

If left untreated during the initial few hours, it becomes life-threatening which results in high morbidity and mortality. It is a dreaded clinical entity caused by a fungus of the order *Mucorales*. It is also known as zygomycosis, usually harmless to a healthy person but fatal in immunocompromised. Most common causative organism is *Rhizopus* species [3]. Diagnosis is by histopathological examination and it is treated by aggressive surgical debridement and appropriate systemic antifungal treatment. It is very rarely found in the neck region and such a case has not yet been reported in literature.

## Case Report

Forty-three year old male, a local politician, initially presented to the emergency department with complaints of right sided earache, ear discharge and right facial palsy. He also had right sided tender neck swelling and oedema extending up to the cheek. Ear examination under microscope showed edema of external canal with perforated tympanic membrane. There was edema of the post aural region and the neck which was tender. He gave history of insect entry into his right ear 2 weeks back which was removed on the same day at a local hospital. He initially had a little discomfort in the right ear and later developed earache which he controlled by self-medication. He is a known diabetic with uncontrolled sugars and not on any proper diabetic regimen. WBC counts and serum creatinine levels were elevated.

MRI imaging done elsewhere showed right mastoiditis and sigmoid sinus thrombosis extending to IJV with features of myositis and cellulitis of neck muscles. There was minimal mucosal thickening in right maxillary and ethmoid sinuses. HRCT Temporal bone was done which showed only minimal mastoiditis without any evidence of bone erosion. Nasal endoscopy and videolaryngoscopy studies were normal.

A Multidisciplinary team meeting was conducted with involvement of neurosurgery, neurology, ENT, endocrinology and infection control departments. Consensus was reached as to defer Mastoid exploration. As per the opinion of infectious disease specialist, the patient was put on empirical high end broad spectrum antibiotics. Ear swab and blood culture was done for him but while awaiting the results, his condition worsened.

A repeat imaging was done and it showed a spreading cellulitis and thrombosis of right internal jugular vein with retropharyngeal space involvement. Neurology review was done and a mastoid exploration with IJV ligation was suggested considering the possibility of Lemierres syndrome. Neck exploration and IJV ligation was done. Subcutaneous tissue showed oedematous fluid and the cervical muscles appeared bulky and oedematous. Few enlarged level 2 and 3 lymph nodes were noted which was sent for histopathological examination. Tracheostomy was done to secure the airway. Thrombosis of IJV was seen extending up to the level of carotid bifurcation. No other specimen was obtained from neck while mastoid exploration revealed partial thrombosis of sigmoid sinus. Mastoid air cells showed moderate amount of granulations.

Post operatively after initial 72 h of improvement, his condition again deteriorated with spreading facial cellulitis, chemosis of right eye and ophthalmoplegia. Repeat imaging was done which was consistent with right orbital cellulitis and retropharyngeal cellulitis without any collection. Videolaryngoscopy showed significant edema of supraglottis and glottis. At this point all microbiological profiles were negative and immunological work up was initiated. Hematology consultation was sought in view of total count reaching 49,000.

**Fig. 1:**
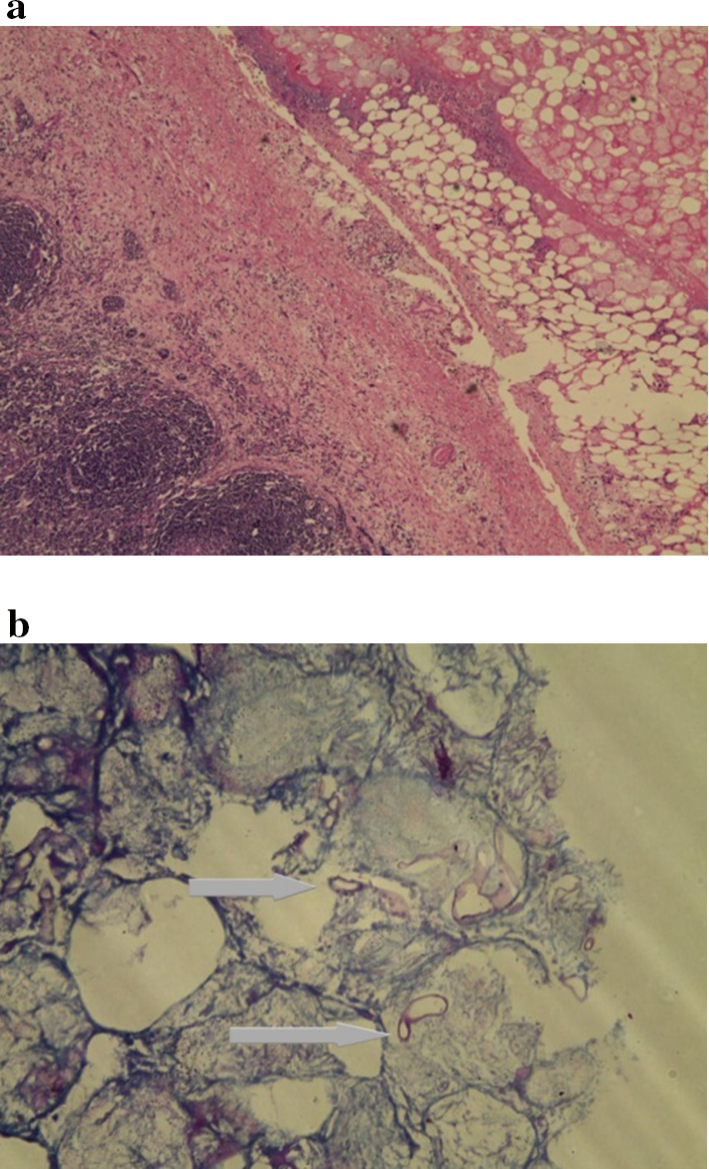
**a** Sections of lymphnode showing follicular hyperplasia, sinus histiocytosis and paracortical expansion with perinodal tissue showing fat with necrosis and dense infiltrate of neutrophils. **b** Amidst the necrotic tissue the arrows showing broad aseptate fungal hyphae with obtuse angle branching seen

The histopathology report arrived on 4th day, surprisingly showing mucor in the peripheral rim of fat tissue around lymph node with features of necrosis in periphery (Fig. 1).

Hence the diagnosis of invasive mucormycosis of neck was reached. Parenteral liposomal amphotericin B 5 mg/kg/day along with oral posaconazole 4 × 200 mg/kg/dose were started [4]. Extensive surgical debridement and antifungal therapy being the only option, there was a dilemma as to how to debride the neck further. In view of the new diagnosis and the anticipated difficulties of further debridement and diabetic nephropathy, a multidisciplinary team meeting was organized with all the participating medical team and the patient’s family members.

**Fig. 2:**
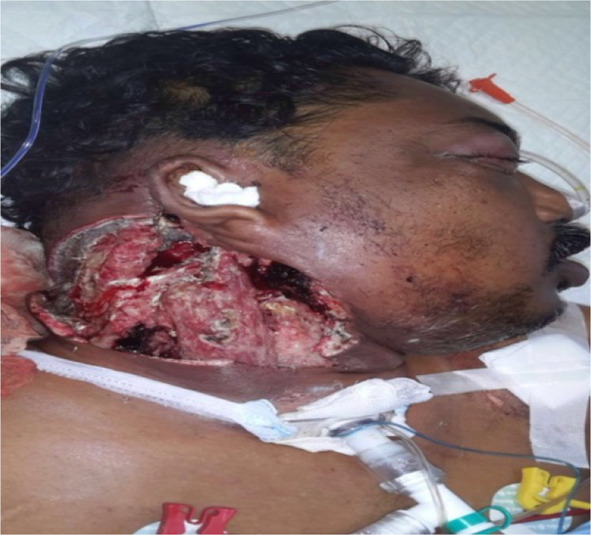
Post neck exploration 3rd day with wound showing necrotic debri in the subcutaneous and deep planes

**Fig. 3:**
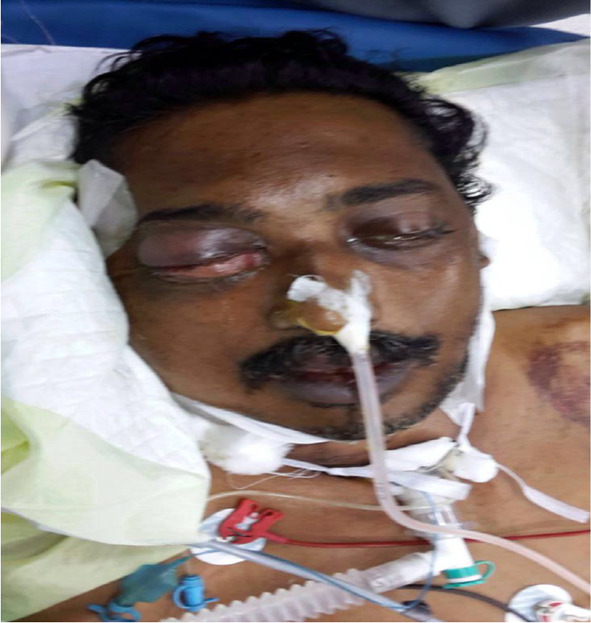
Post neck exploration day 3 with patient developing right orbital ecchymosis and proptosis

The neck wound was showing features of necrosis along with vision deteriorating thus a need for orbital exenteration was discussed with the patient and the family (Figs. 2, 3).

The neck wound was re explored, but the entire picture was different from earlier. There was now extensive necrosis of cervical muscles, parotid & submandibular gland. A third Multidisciplinary team meeting was called for, headed by ENT which included Ophthalmology, Head and neck, Neurosurgery, Plastic surgery and Anesthesia specialities. Another surgical debridement of the wound with orbital exenteration was done.

Staged debridements under local anaesthesia were continued till disease free limits were attained. Since it was difficult to attain disease free vertical depth beneath the mastoid, the endoscopic skull base team was called upon and they came up with the innovative idea of using microdebrider in deep wound. The debridement was carried out till atlas and around the foramen magnum which was the end point of dissection.

Multiple challenges were encountered post operatively including urinary sepsis with septicemia, high volume diarrhoea with impending shock and acute renal failure. He was managed in ICU under the care of Intensivist with strong support from nephrology, urology & gastroenterology teams. He was given tigecycline during the course of his treatment as he developed multidrug resistant Klebsiella pneumonia, following which he developed tigecycline induced arrhythmia and cardiac arrest which was treated by cardiology team. He underwent feeding jejunostomy for long term nutritional requirements.

**Fig. 4:**
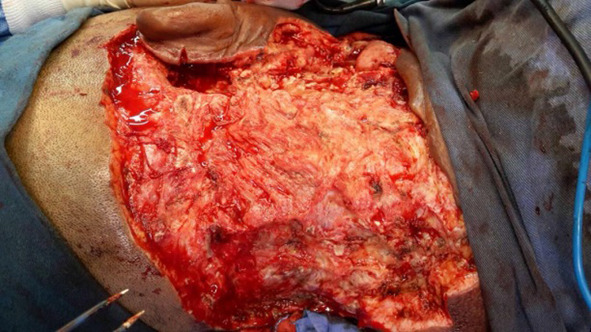
Post debridement attained disease free margins

**Fig. 5:**
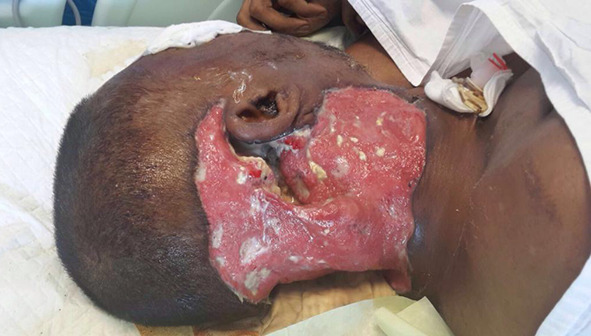
Healing happening by granulation

Finally after 3 weeks, reasonably good dry granular superficial wound without microbial activity was attained which was confirmed by repeated swab cultures (Figs. 4, 5).

**Fig. 6:**
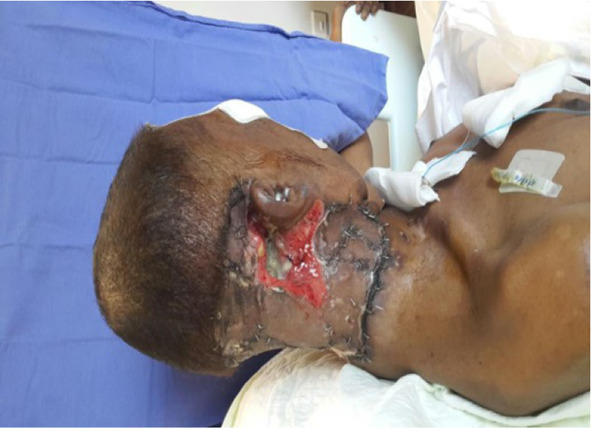
Post skin grafting

Deep wound got better, started healing by granulation tissue formation. Skin grafting of the superficial neck wound was done by plastic surgeon after the deep wound got granulated completely (Fig. 6).

**Fig. 7:**
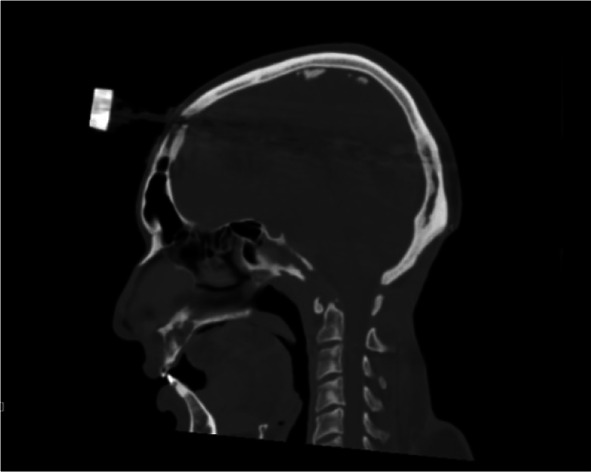
Post debridement CT showing eroded skullbase and atlas

**Fig. 8:**
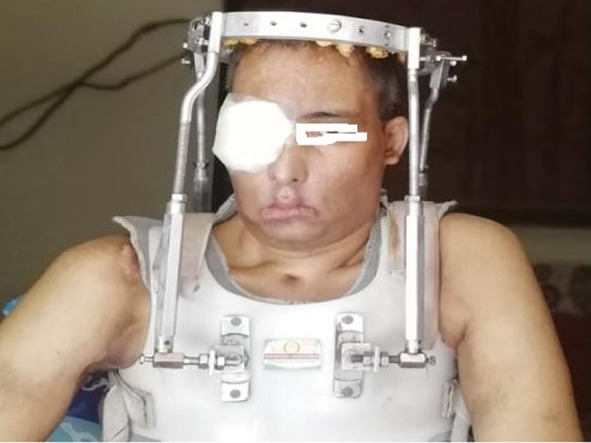
Post treatment patient on halo-splint

With devoted effort of physiotherapy team and a halo splint supporting his cervical spine, he is now walking with the help of a walker and is able to perform his basic daily chores (Figs. 7, 8).

## Discussion

The inoculation of fungus during the FB entry into the ear could have been the route of introduction of fungus. Major predisposing factor for the patient was uncontrolled diabetes due to neglected glycemic control.

The expected routes of spread are subcutaneous tissues and vascular channels of EAC via fissures of santorini then to the parotid gland, retromandibular vein, internal jugular vein and then to the sigmoid sinus, through which neck spaces and cervical muscles got involved. Another route could be facial vein, orbital space, soft tissue and neck muscles.

Since patient had diabetic nephropathy and renal parameters were getting affected during administration of liposomal amphotericin and liver enzymes were getting altered with posaconale, we used supplementation of local amphotericin while doing daily dressing. On reviewing the literature, the use of hydrogen peroxide in open wounds with mucor was documented [5]. So it was innovatively tried and topical application of amphotericin gel along with hydrogen peroxide was done over the remaining dark necrotic tissue after each debridement. Along with this, application of chemical debrider with papain & urea was done over the slough covered areas. Topical antibioics including colloidal silver were used for dressing and it was changed periodically in order to reduce antibiotic resistance. Silver has been proved to be useful prophylactic and therapeutic agent for the prevention of wound colonization by organisms that impede healing, including antibiotic-resistant bacteria [6]. The dressings were changed twice every day till healthy granulation tissue was seen all around.

Once disease was controlled locally, halo splint was used for stabilising the neck as the vertebrae was already partially destroyed by the disease and debridement. Swallowing therapy was initiated later and feeding gastrostomy was removed once he was able to swallow adequately.

The differential diagnosis for this case included otitis media with neck abscess, skullbase osteomyelitis lemierre syndrome etc. Lemierre syndrome is a rare and potentially life-threatening complication of bacterial infections affecting previously-healthy adolescents and young adults. Usually develops in association with a bacterial throat infection, but it may develop in association with an infection involving the ears, salivary glands (parotitis), paranasal sinuses, or teeth [7]. Cutaneous mucormycosis is another differential diagnosis for this which is classified as primary and secondary. In primary disease, the skin is primarily infected, in secondary form usually happens by dissemination from other locations, more commonly from a rhinocerebral infection [8].

## Conclusion

Here is an uncommon presentation of an rare disease. Timely intervention along with innovative treatment methods coupled with team effort has helped save the patient from certain mortality.

## Data Availability

Available in hospital information system.
